# Discovery of Novel Chemotype LRRK2 Inhibitors Through AlphaFold2-Generated Structure-Based Docking Screen

**DOI:** 10.3390/ijms27083391

**Published:** 2026-04-09

**Authors:** Rishiram Baral, Jeong In Lee, Jun-Goo Jee

**Affiliations:** Research Institute of Pharmaceutical Sciences, College of Pharmacy, Kyungpook National University, Daegu 41566, Republic of Korea; rishirambaral1996@gmail.com (R.B.); benzene04@naver.com (J.I.L.)

**Keywords:** AlphaFold2, docking screen, inhibitor, kinase, LRRK2

## Abstract

The structures predicted by AlphaFold can provide unprecedented opportunities for docking screens; however, experimentally validated examples of using the apo-form are limited. This study reports novel chemotype inhibitors targeting the leucine-rich repeat kinase 2 (LRRK2) kinase domain through a docking screen using one of the ensemble structures starting from the template deposited by AlphaFold2. The MODELLER software generated the ensemble. The conformer that showed the best early enrichment of true positives with the mixture of known ligands and their property-matched decoys was selected. The docking screen against approximately 1.3 million small molecules and enzyme-based assays with the LRRK2 kinase domain followed. We selected 17 molecules, excluding those similar to all known kinase inhibitors. Combined with analogs-by-catalog, ten new small molecules with Ki values below 15 μM were discovered, including one sub-μM inhibitor. To test selectivity, enzyme assays with a mutant and six additional kinases, including known off-targets of existing LRRK2 inhibitors, were performed using three inhibitors. The data suggest that the novelty in chemical structure may be insufficient for providing selectivity. Our approach is generally applicable to cases where information on known binders is available but experimental structure is not.

## 1. Introduction

High-resolution protein structures are invaluable tools that provide critical, atomic-level insights into biological function, stability, and mechanism. Furthermore, they can facilitate small-molecule drug discovery. Docking is a well-known application for utilizing three-dimensional (3D) structures [1]. Based on the complementarity of geometry, the computational calculation of poses and energies of the small molecule library can narrow down the molecules that likely bind to targets. Identifying the structure–activity relationship backed by structural information is a mandatory step for reliable data interpretation and activity enhancement [2].

Not all structures are helpful for docking. Most docking algorithms assume the starting structure as a rigid body. However, subtle changes in the geometry of the active site can result in a significant deviation in poses and energies, thereby worsening the enrichment of true positives [3]. To overcome the drawbacks caused by the assumption of a rigid body, induced-fit algorithms have been developed. Nevertheless, they are currently not suited for high-throughput docking due to their enormous computational time [4]. One possible way to overcome this weakness is to use an ensemble of protein structures with different conformations. Subsequently, one can select the most suitable conformation for the docking from the ensemble [5,6].

AlphaFold2 and AlphaFold3 are revolutionary achievements in structural biology [7,8,9]. The accuracy of the predicted model is comparable to that of crystallography. This is particularly critical when no homologous protein structures have been determined experimentally. Despite the widespread application of AlphaFold2-predicted structures, their direct utility in prospective large-scale structure-based virtual screening (SBVS) campaigns remains limited by the paucity of experimentally validated examples. Here, we report the successful application of an AlphaFold2-derived ensemble model to discover novel chemotype leucine-rich repeat kinase 2 (LRRK2) inhibitors with sub-micromolar potency, providing one of the few prospective demonstrations that predicted apo-structures can independently support hit discovery for a target.

We selected the LRRK2 kinase domain as a target for the docking screen. Although LRRK2 is a massive 2527-amino acid protein whose inhibition is a validated strategy for treating Parkinson’s disease [10], high-resolution structures of its primary druggable kinase domain were unavailable at the inception of this study. The kinase domain corresponds to residues 1879–2138. Although a few cryo-EM structures of full-length LRRK2 have been reported, their resolution remains insufficient to define the binding pocket with atomic precision [11,12]. Therefore, the LRRK2 kinase domain represents a compelling test case for AlphaFold2-based virtual screening: a pharmaceutically important target for which sufficient ligand activity data exist to benchmark docking performance, yet complex structures at a resolution suitable for structure-based drug design are limited.

So far, most atomistic interpretations of the intermolecular interactions between the LRRK2 kinase domain and its inhibitors have relied on the structures of either ROCO4, the LRRK2 ortholog from *Dictyostelium discoideum* (*D. discoideum*) [13,14], or surrogate kinase domains [14,15]. More than seven structures of the ROCO4 kinase domain are available in complex with small molecules. The structures of CHK1 10-point mutants with LRRK2 inhibitors have also been reported. However, the sequence similarities between the LRRK2 kinase domain and the ROCO4 and surrogate kinase domains are limited. The ROCO4 kinase domain shares 28% sequence identity with that of LRRK2, and the sequence of the glycine loop differs between LRRK2 and the CHK1 10-point mutant [16]. Notably, although ROCO4 has been used as a surrogate for the LRRK2 kinase domain, global sequence alignment reveals clear differences at key ligand-binding residues, such as Ala1950, Ser1951, and Arg1957 [17]. These differences, although sometimes chemically conservative, may affect the local binding geometry. Therefore, we constructed and used an AlphaFold2-derived model to better capture the target-specific binding sites for virtual screening.

Gain-of-function mutations in LRRK2, most notably G2019S, are the most common genetic cause of familial Parkinson’s disease [18]. The mutation enhances kinase activity and impairs lysosomal function, providing a genetic rationale for kinase inhibition as a disease-modifying strategy. Several potent kinase domain inhibitors have been developed and are undergoing clinical trials. The inhibitors include MLi-2 (IC_50_ = 0.76 nM) [19], PF-06447475 (IC_50_ = 3 nM) [20], and GNE-0877 (Ki = 0.7 nM) [21], and BIIB122 (DNL151) that is currently evaluated in a Phase 2 clinical trial [22]. However, most reported inhibitors share common scaffolds, such as aminopyrimidine, indazole, and pyrrolopyrimidine, highlighting the need for new chemical series.

**Figure 1:**
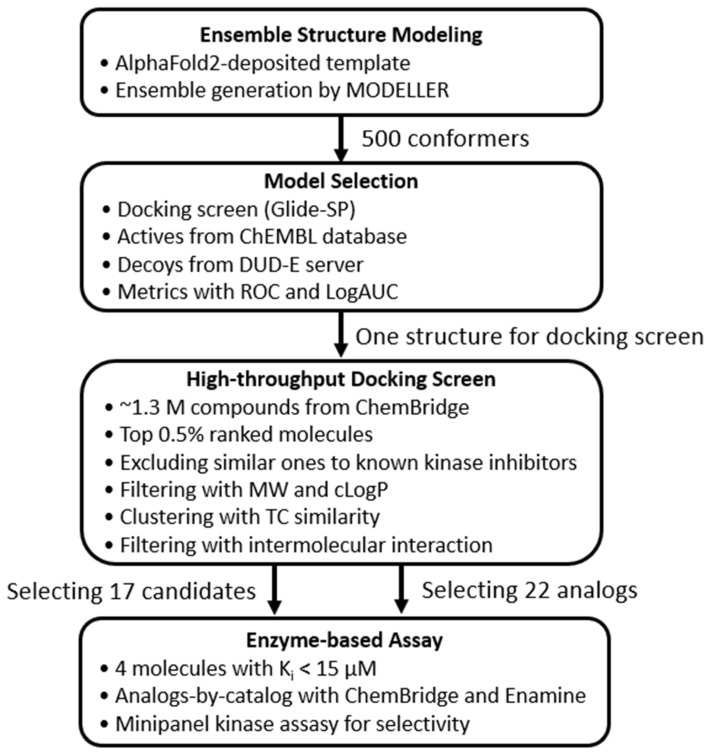
Workflow of virtual screening and experimental validation pipeline. The AlphaFold2-predicted LRRK2 kinase domain structure was used as a template for MODELLER-based ensemble generation (500 lower-scoring conformers of 3000 generated). The optimal conformer was selected by benchmarking enrichment with known inhibitors and property-matched decoys, followed by high-throughput docking of approximately 1.3 million compounds. Hit selection, enzyme-based assays, analog expansion, and selectivity profiling were performed.

This study aimed to demonstrate that an AlphaFold2-derived LRRK2 kinase domain structure, refined via ensemble modeling, can be effectively used for virtual screening to discover novel inhibitors. We aimed to identify compounds with low chemical similarity to known kinase inhibitors and validate their activities through enzyme-based assays, thereby evaluating both the feasibility and limitations of AlphaFold2-based SBVS for pharmaceutically relevant targets lacking crystal structures. Figure 1 shows an overview of the workflow.

## 2. Results

### 2.1. Homologous Structures Enabled the Identification of LRRK2 Residues Potentially Significant for Interacting with Inhibitors

We extracted the region corresponding to the kinase domain (1879–2138) from the AlphaFold2-generated full-length structure of human LRRK2 (UniProt ID: Q5S007) [7,8]. We subsequently compared the structure with known crystal structures using the DALI server [23]. The retrieved structures with the highest Z-scores were SYK (PDB code: 6ZCU-A, Z-score: 30.3, sequence identity: 27%), followed by FES (3CD3-A, 30.2, 25%), ROCO4 (4YZN-A, 29.9, 28%), CTR1 (3PPZ-A, 29.7, 31%), CDC42 (6VQM-A, 29.5, 28%), SRC (3DQW-C, 29.1, 27%), EPHA2 (4TRL-A, 29.0, 22%), ABL (2V7A-A, 28.9, 24%), DAPK (5AUW-A, 28.9, 26%), and BRAF (4E26-A, 28.8, 28%). It is worth noting that even ROCO4, an ortholog of LRRK2 from *D. discoideum*, has a limited sequence identity of 28%. For SYK, several structures in complex with small molecules besides 6ZCU-A were ranked highly, including 6ZCR-A (Z-score: 30.3), 3VF8-A (30.2), 4PX6-A (30.2), 5CXZ-A (30.2), and 1XBC-A (30.2).

These homologous templates provided critical structural clues for identifying potential inhibitor-contacting residues. For example, hydrophilic or specific interactions between SYK and its inhibitor 5JG occur at Ala451, Glu452, Lys458, Arg498, and Asn499 in the 6ZCU-A coordinates. The corresponding residues in LRRK2 are Ala1950, Ser1951, Arg1957, His1998, and Asn1999. Among these, Ala1950 and Asn1999 are conserved in residue type and are thus predicted to serve as contacting residues. Considering the intermolecular interaction information, we selected the five most overlapping residues of Lys1906, Glu1948, Ala1950, Asn1999, and Asp2017 in LRRK2 as the putative contact sites for the inhibitors. We used this information to filter out unlikely molecules during the post-docking stage.

### 2.2. MODELLER-Generated Ensemble Included Conformers Showing Better Metrics than the AlphaFold2-Generated Template Structure

The MODELLER-generated structural ensemble exhibited low overall conformational variance. The root–mean–square deviation of the backbone atoms (RMSD) in the 500 structures against the AlphaFold2 structure was 0.228 ± 0.034 Å. We tested whether the ensemble contained a better conformer that was more suitable for the docking screen. Logarithmically scaled area under the curve (AUC) (LogAUC) [24] was used as the main metric derived from the receiver operating characteristic curve (ROC) to emphasize the earlier enrichment of true positives over false positives.

We extracted LRRK2 inhibitors from the ChEMBL database [25]. The strongest 60 molecules in terms of pChEMBL value and molecular weights under 500 Da were identified. To ensure chemical diversity, we selected 36 non-redundant molecules by excluding those with a Tanimoto coefficient (TC) ≥ 0.6 relative to more potent compounds. The TC 0.6 threshold is commonly applied in chemotype clustering and scaffold diversity analyses to partition compound libraries into structurally distinct clusters [26,27,28]. The chemical structures of the 36 molecules are shown in Figure S1. The DUD-E server [29] generated physicochemically matched but topologically different decoys. Subsequently, the AUC and LogAUC were calculated from the test docking simulation results with ligands and decoys.

**Figure 2:**
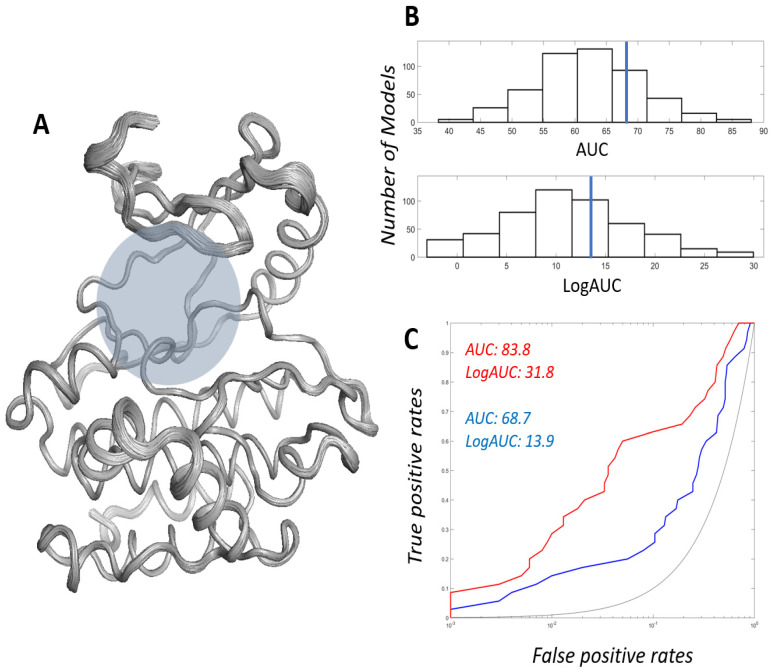
Pre-docking evaluation of the AlphaFold2-predicted structure and the MODELLER-derived ensemble. (**A**) Structural ensemble consisting of the 500 MODELLER-derived models aligned to the AlphaFold2-predicted LRRK2 kinase domain. The approximate binding site region is indicated by a gray circle. (**B**) Distribution of AUC (**top**) and LogAUC (**bottom**) values across 500 ensemble models. The values for the AlphaFold2-predicted template are indicated (blue). (**C**) Representative ROC curves from the AlphaFold2 template (blue) and the selected conformer 0360 (red), with random enrichment shown in gray.

The histograms of the distributions of AUC and LogAUC are presented in Figure 2. The average values were 62.2 ± 8.3 and 11.2 ± 6.6 for AUC and LogAUC, respectively. The AlphaFold2-derived template structure showed slightly better metrics than the average values, with 68.7 and 13.9. We selected the 0360 conformer as the best structure for the docking screen. The model had AUC and LogAUC values of 83.8 and 31.8, respectively. A comparison of the profile with those from the AlphaFold2 template demonstrated distinct improvement despite slight structural differences (Figure 2). This again supports the idea that subtle structural differences can generate different enrichments of true positives. Here, it is noteworthy that a higher enrichment of true positives does not necessarily imply lower energies for true positives. Rather, it reflects the difference in energies between true positives and false positives. Indeed, the mean Glide-SP energy for the 36 true positives was −7.860, ranking 0360 17th. In contrast, the ranking of 0360 was second in terms of energy differences between true and false positives.

### 2.3. High-Throughput Docking Screen and Enzyme-Based Assay Resulted in Novel Chemotype Inhibitors

Following the high-throughput docking screen, a series of steps for selecting candidates was performed using the top 0.5% ranked molecules. First, chemicals similar to any known kinase inhibitors, including LRRK2 inhibitors, with a higher TC than 0.35 were excluded. We considered approximately 97,000 ChEMBL-registered inhibitors from 443 kinases with Ki or IC_50_ values below 10 μM. Molecules with molecular weights > 400 Da or a calculated logP > 4.0 were excluded. Although pre-docking filtration of the candidate library would have reduced computational time, we retained the full dataset to comprehensively analyze the chemical scaffolds enriched during the docking process. Second, the resulting structures were clustered using a cut-off of TC = 0.5 [30]. The lowest-energy structure was selected as the representative of each cluster. Third, we checked for the existence of intermolecular contacts through residues that we defined as key residues, excluding those with fewer than two contacts. Fourth, we manually checked the geometry of the intermolecular interactions for hydrogen bonds or salt bridges and implausible intramolecular conformations [3]. Finally, 17 molecules were selected and purchased for experimental testing (Figure S2).

**Figure 3:**
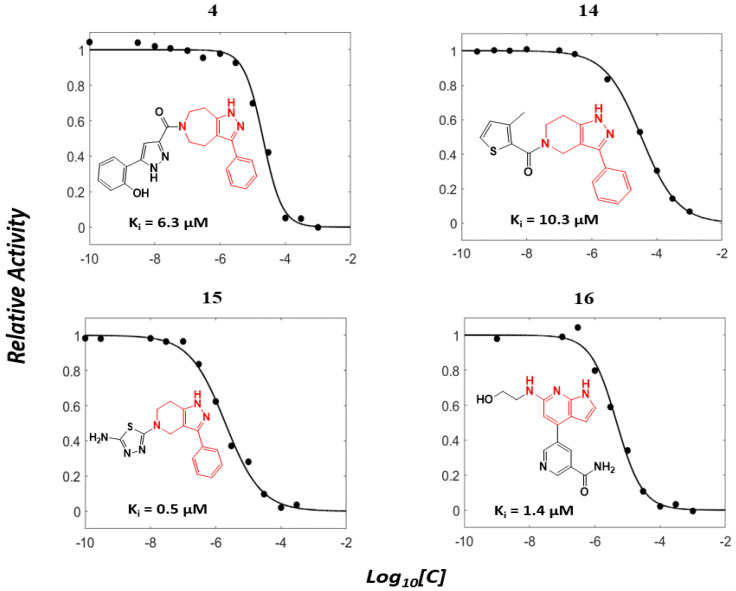
Concentration-dependent inhibitory profiles of hit compounds. Dose–response curves for compounds **4**, **14**, **15**, and **16** are shown. Kinase activity was normalized to a relative scale (0–1), and Ki values were estimated by nonlinear regression. The predicted hinge-binding moieties of each compound are highlighted in red.

Enzyme-based assays revealed that four of the tested molecules showed a noticeable decrease in LRRK2 kinase activity at a single concentration of 50 μM (Figure S3). Four molecules, **4** (Vendor ID: 56275466), **14** (29661093), **15** (21393542), and **16** (99611978), exhibited Ki values below 15 μM. Among the hits, **4**, **14**, and **15** shared similar chemical moieties. Compounds **14** and **15** possess phenyl-pyrazolopyridine, whereas **4** contains phenyl-pyrazoloazepine. Compound **16** contains an amino-pyrrolopyridine moiety. Interestingly, however, the presence of key scaffolds was insufficient to inhibit LRRK2 activity. Molecules **1**, **2**, **5**, **7**, **10**, and **16** possess amino-pyrrolopyridine as a hinge (Glu1948 and Ala1950) interacting moiety (Figure S2); however, only **16** exhibited inhibitory activity. Similarly, compounds **3**, **6**, **8**, **13**, **14**, and **15** contain pyrazolopyridine that interacted with the hinge region (Figure S2). However, only **14** and **15** exhibited inhibitory activity. The lack of inhibitory activity in the remaining compounds underscores that the hinge-binding motif alone is insufficient for LRRK2 inhibition, highlighting the necessity of experimental validation of computationally predicted hits. Profiling of concentration-dependent inhibitory activities quantified the level of inhibition (Figure 3 and Table 1). Remarkably, compound **15** exhibited a Ki of 530 nM despite its low molecular weight of 298 Da. For comparison, the known LRRK2 inhibitor MLi-2 showed a Ki value of 1.4 nM, consistent with the previous result within uncertainty [19], confirming the reliability of our enzyme-based assay. It should be noted that the activities were measured with a mild detergent (0.01% Triton X-100) to minimize aggregation-mediated effects of false positives [31], and there was no detectable interference in the ADP-Glo detection controls.

### 2.4. Cheminformatics Supports the Novelty of the Hits

**Figure 4:**
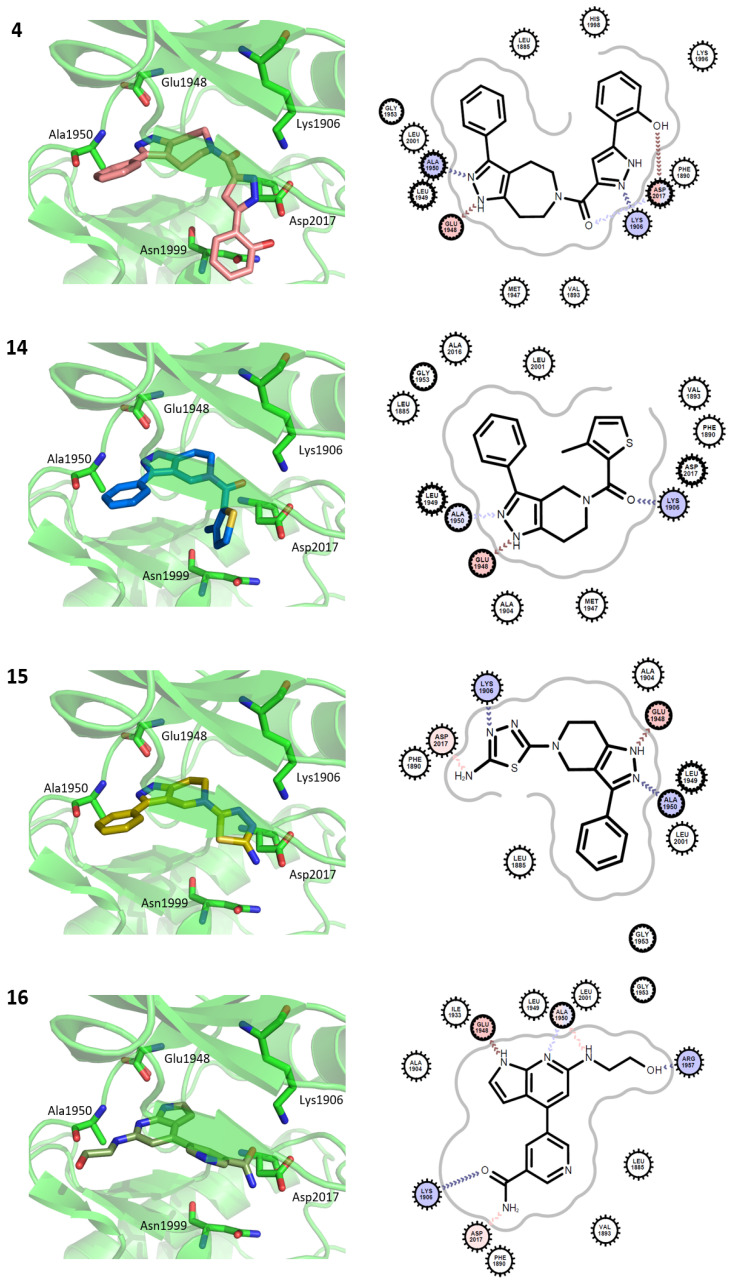
Docked poses and binding interactions of active hit compounds. The top four hit compounds (**4**, **14**, **15**, and **16**) are shown with their docked poses (**left**) and 2D interaction diagrams (**right**). Key binding site residues are visualized as sticks in the 3D structures. Two-dimensional diagrams were generated using the OpenEye toolkit, highlighting hydrogen bond donors (blue) and acceptors (red). Contacting residues are labeled in circles, with inward or outward lines indicating interaction with the main chain or side chain, respectively.

The binding poses of the four molecules predicted by docking are shown in Figure 4. Intermolecular hydrophilic contacts include hydrogen bonds with two residues, such as Glu1948 and Ala1950, in the hinge region of all molecules. Hydrophilic interactions were observed between Lys1906, Arg1957, and Asp2017. Importantly, these interactions were predicted from homologous structures a priori. The binding mode comparison can explain the more potent inhibition of **15** compared to **14**. The amino-thiadiazole part of **15** can form two hydrogen bonds with Lys1906 and Asp2017. Conversely, the hydrogen bond through Lys1906 disappears in the methyl-thienyl-methanone of compound **14**. The closest known LRRK2 inhibitors for **4**, **14**, **15**, and **16** are CHEMBL3651748, CHEMBL2403368, CHEMBL4060479, and CHEMBL2333139, with TC values of 0.282, 0.273, 0.262, and 0.333, respectively (Table 1). In particular, **16** shares only limited similarity to CHEMBL2333139, despite having the highest TC, supporting the need for visual inspection. Given this dissimilarity, together with the distinct binding poses and interaction patterns shown in Figure 4, it is unlikely that **16** and CHEMBL2333139 share similar binding modes.

**Figure 5:**
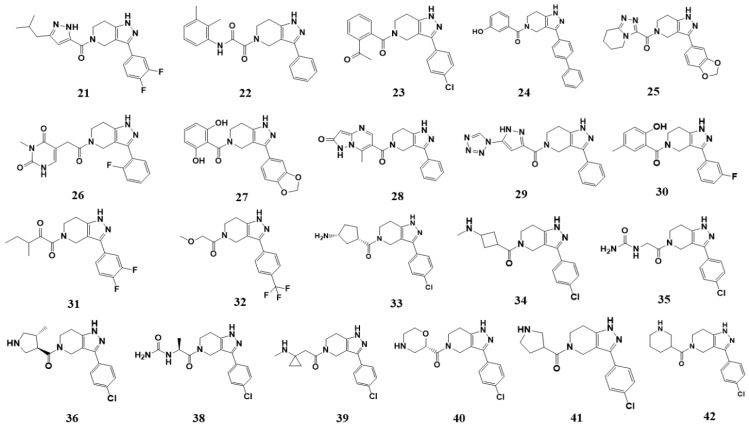
Chemical structures of analogs-by-catalog selected based on compound **15**. A total of 22 commercially available analogs structurally related to hit compound **15** were selected for follow-up testing. The core scaffold was preserved, while variations were introduced at peripheral positions.

**Figure 6:**
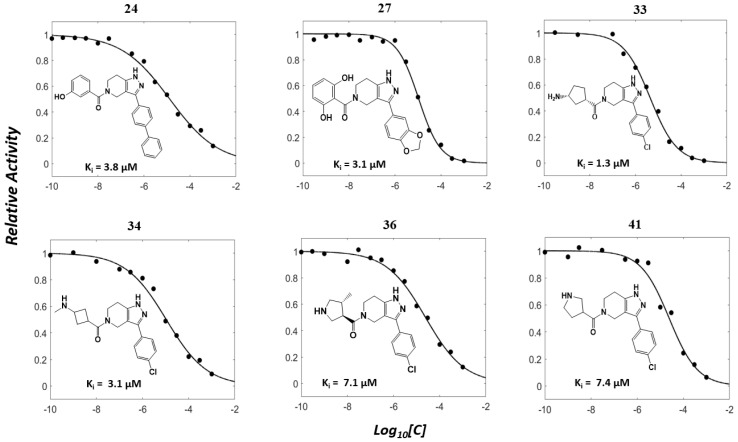
Concentration–response profiles of analogs derived from compound **15**. Dose–response curves of six analogs (**24**, **27**, **33**, **34**, **36**, and **41**), identified from a catalog-based analog search initiated from compound **15**, are shown. Each compound was evaluated for LRRK2 kinase inhibition. Relative kinase activities were normalized from 0 to 1 and plotted against the logarithm of compound concentration. The calculated Ki values are shown within each plot alongside the corresponding chemical structures.

Based on the presence of phenyl-pyrazolopyridine in the most potent inhibitor **15**, we searched for commercially available phenyl-pyrazolopyridine-containing molecules from the ChemBridge and Enamine databases. Molecular docking simulations of the retrieved molecules were performed and the binding modes were compared to that of **15**, resulting in 22 analogs for testing. The molecules and their quantified inhibitory activities against the LRRK2 kinase domain are presented in Figure 5 and Figure 6 and in Table 2. No molecule was more potent than **15**, whereas six molecules, including **24** (Ki = 3.8 μM), **27** (3.1 μM), **33** (1.3 μM), **34** (3.1 μM), **36** (7.1 μM), and **41** (7.1 μM), exhibited Ki values less than 10 μM (Figure 6 and Table 2).

Understanding the physicochemical properties and structure–activity relationship (SAR) between structurally related molecules is important for drug discovery. We calculated the properties, including blood–brain barrier penetration possibility, using the SwissADME web server for all the molecules [32]; however, we could not establish any apparent rules (Supporting Materials Table S1). Notably, the BOILED-Egg model predicted that compound **15**, with a topological polar surface area (TPSA) of 101 Å^2^, falls outside the BBB-permeant region, suggesting that reducing TPSA while maintaining potency would be a priority for future optimization targeting CNS indications.

We also performed an SAR analysis on 23 molecules (compounds **14**, **15**, and **21**–**42**); however, we could not find statistically significant terms, mainly because of the small number of analogs. Therefore, we applied the latest free energy calculations using Boltz-2 [33]. Remarkably, the calculated energies by Boltz-2 and the energies converted from Ki values showed a correlation factor with R = 0.609 (Figure S4). Statistical analysis of the data indicates that the energies calculated by Boltz-2 and the experimental Ki values are correlated, with *p*-value of 2.1 × 10^−3^. This result suggests the possibility of designing more potent inhibitors through Boltz-2 calculations before chemical synthesis.

### 2.5. Enzyme-Based Assays with Other Kinases Showed Off-Target Inhibition

We tested several proteins to determine the selectivity of these inhibitors against them. LRRK2 (G2019S), a mutant believed to cause Parkinson’s disease, was included. To rigorously evaluate whether our novel chemotypes successfully circumvent the selectivity issue often found in kinase inhibitors, we profiled our hits against a curated panel of off-target kinases associated with LRRK2 inhibition. We chose the known off-target kinases of existing inhibitors with IC_50_ or Ki values below 500 nM. The kinases were MST1 (UniProt ID: Q49A61) for PF-06447475 [20], TTK (P33981) for GNE-0877 [21], and ASK1 (Q99683), CLK2 (P49759), and NIK (Q99558) for MLi-2 [19]. Furthermore, SYK (P43405) was included to check the potential off-target effects that may be generated by structural similarity. As described in Section 2.1, SYK showed the highest Z-score against the LRRK2 kinase domain in the comparison with the AlphaFold2-predicted structure. The selectivity was tested against these seven kinases using the three most potent molecules: **15**, **16**, and **33**.

The enzyme-based quantification of the inhibition is shown in Table 3 and Figure S5. All of these were inhibitors of LRRK2 (G2019S). This equipotent inhibition of the G2019S mutant is consistent with the location of the Gly2019 residue within the DFG motif, which is distal to the hinge-binding region where our inhibitors are predicted to interact (Figure 4). Interestingly, **15** was equally potent against CLK2 and TTK, **16** comparably inhibited TTK and ASK1, and **33** was more potent in inhibiting MST1. However, no molecule strongly inhibited SYK. Of note, the off-target profiles of **15** and **33** showed different patterns, although **33** is an analog of **15**.

We also prepared complex models of compound **15** with CLK2 and TTK to investigate the origin of the strong inhibition, using the same method as for LRRK2. We then compared their poses and intermolecular interactions. The sequence identities in the kinase domains of CLK2, TTK, and LRRK2 are limited. The values for CLK2 and LRRK2, and for TTK and LRRK2, were 24% and 22%, respectively. Nevertheless, we observed that all poses were similar to one another and all shared key contacts (Figure S6). Of the four key hydrophilic contacts from the residues of Lys1906, Glu1948, and Ala1950, and Asp2017 in LRRK2, three contacts from Lys1906, Glu1948, and Ala1950 existed for all three cases. In contrast, an interaction through Asp2017 was observed in CLK2. The sharing of the poses and contacts may be insufficient to explain the strong inhibition; however, it will serve as a starting point for developing selective inhibitors.

Another meaningful question is whether these off-targets can be predicted using existing cheminformatics tools. To predict the potential targets of **15**, **16**, and **33**, we used the Similarity Ensemble Approach (SEA) server [34,35]. SEA relates proteins to one another by comparing the set-wise chemical similarity among their ligands rather than by protein sequence or structure. For a given query molecule, SEA calculates the sum of pairwise Tanimoto coefficients between the query and all known ligands of each target protein. This raw similarity sum is then compared with a null distribution derived from randomly related molecules, modeled by an extreme value distribution (EVD), to yield a *p*-value. The *p*-value represents the probability that the observed cumulative similarity between the query and a target’s ligand set would arise by chance alone; thus, a lower *p*-value indicates a higher likelihood that the query molecule shares pharmacological activity with that target’s known ligands [34,35]. The absence of predicted targets with a *p*-value < 10^−15^ for compound **15** further substantiates its structural novelty (Figure S7). Those that showed a *p*-value of <10^−15^ were all non-kinase proteins for **16** (Figure S7). The proteins were nicotinamide N-methyltransferase, NAD-dependent protein deacetylase sirtuin-3 and -2, and hepatocyte growth factor-like protein. For compound **33**, the four retrieved targets included two kinases, mitogen-activated protein kinases 11 and 4 (Figure S7). The other two were retinol-binding protein 4 and potassium channel subfamily K member 3. Strikingly, none of the SEA-predicted targets included LRRK2, which was more strongly inhibited in experiments. This supports the significance of experimental validation. Additionally, our data may indicate that examining the activity against off-targets of known inhibitors can be a simple test for examining the off-targets of new inhibitors.

## 3. Discussion

The strength of the AlphaFold2-generated structure lies in its accuracy, at least in the region of the backbone and χ^1^ angles. This feature may narrow the conformational space that needs to be sampled, facilitating the identification of a suitable conformation for docking. In this study, the MODELLER-generated 500 conformers included the proper structures. An inaccurate initial template may require a more diverse ensemble. In such cases, alternative conformational sampling methods, including the Rosetta relax algorithm [36], molecular dynamics simulations [5], or elastic network models [37], could be employed. An interesting piece of information is the differences between the ensembles generated by AlphaFold2 and MODELLER. We prepared an AlphaFold2 ensemble consisting of 50 structures by adapting the ColabFold method and changing the random seed [38]. We found that the mean RMSD of the ensemble was 0.578, which was approximately 0.35 higher than that of MODELLER (see Section 2.2). This indicates that the MODELLER ensemble places greater emphasis on side-chain conformation.

Several recent studies have explored AlphaFold2-predicted structures for virtual screening with varying degrees of success. Retrospective benchmarks have shown that AlphaFold2 models generally underperform experimental structures in docking evaluations [39,40]. Nevertheless, prospective studies have yielded experimentally validated results. Lyu et al. docked large compound libraries against unrefined AlphaFold2 models of the σ2 and 5-HT2A receptors and achieved hit rates comparable to those obtained from experimental structures [41]. Ren et al. combined the AlphaFold2 structure of CDK20 with AI-driven molecular generation to discover a nanomolar inhibitor (IC_50_ = 33 nM) [42]. Baselious et al. identified a selective HDAC11 inhibitor (IC_50_ = 3.5 μM) through docking on an optimized AlphaFold2 model [43]. Díaz-Holguín et al. screened over 16 million compounds against an AlphaFold2 model of TAAR1, achieving a 60% hit rate that outperformed a homology model [44]. Our approach shares the ensemble strategy with the TAAR1 study but differs in that we generated conformational diversity using MODELLER from the AlphaFold2 template and selected the optimal conformer through systematic benchmarking with known inhibitors and property-matched decoys. Importantly, whereas most previous studies targeted proteins with closely related crystal structures, the LRRK2 kinase domain had a limited sequence identity (≤31%) to all structurally characterized kinases at that time. Despite this challenge, our screen identified a sub-micromolar inhibitor (Ki = 530 nM) with a novel chemotype (TC < 0.3), demonstrating that AlphaFold2-derived ensemble models can support hit discovery, even for structurally undercharacterized targets.

AlphaFold2 appears to be unable to generate alternative or minor conformations from sequence alone, even when the stand-alone program is executed with the casp14 option. The calculated models were too close to each other in the ATP-binding region, all revealing a type I conformation in the DFGin-BLAminus loop (Asp2017, Tyr2018, and Gly2019) [45]. Type I conformation is the most common 3D kinase conformation, occupying more than 80% of the known kinase structures. Therefore, ensemble structures starting from the AlphaFold2 template also have a type I conformation. This implies that the discovery of type II inhibitors using our ensemble and rigid-body docking protocol is challenging. Starting with a surrogate model developed based on type II kinase structures is a more appealing strategy, as attempted by Song et al. with template structures and AlphaFold2 [46].

Another key question is how the AlphaFold2 template differs from the MODELLER-generated structure and what effect this difference has on the docking outcomes. Although the backbone root-mean-square deviation (RMSD) between the AlphaFold2 template and the selected SBVS conformer (0360) was only 0.23 Å, the two coordinates exhibited markedly different early enrichment profiles. We docked the hit molecules (**4**, **14**, **15**, and **16**) into the AlphaFold2 template and compared their poses with those in this study. The poses of **14** and **15**, the two molecules sharing scaffold similarity, were completely overlapped. However, **4** and **16** exhibited slightly different poses and lacked intermolecular hydrophilic interactions with Lys1906 (Figure S8). Visual inspection revealed that the side-chain conformations of Lys1906 and Leu2001 were slightly altered between the two structures. Lys1906 and Leu2001 likely play roles in intermolecular hydrophilic and hydrophobic interactions. Our data demonstrate that the use of ensemble structures improves two key factors in the predocking stage [3,47]: enrichment and pose reproduction. The chosen structure also influences pose reproducibility, which cannot be judged a priori because of the lack of a complex structure.

After our virtual screening and experimental validation had been completed, near-atomic-resolution cryo-EM structures of the LRRK2 kinase domain in complex with type I and type II inhibitors were reported [17,48]. These independently determined structures provide a rigorous post hoc assessment of our computational models. The superposition of conformer 0360 and cryo-EM LRRK2 structures with type I inhibitors (PDB IDs: 8FO7, 8U7H, and 8TXZ) yielded pairwise backbone RMSD values in the range of 1.15–1.32 Å (Figure S9). Interestingly, the side-chain conformations of Lys1906 and Leu2001 in the cryo-EM structures are closer to those of the AlphaFold2 template than to those of conformer 0360. This indicates that AlphaFold2 predicted these binding-site residues with remarkable accuracy. It also highlights an important practical aspect of ensemble-based virtual screening: the conformer yielding the best enrichment in benchmarking does not necessarily possess the most physically accurate side-chain geometry. A similar observation was reported by Lyu et al. [41], who found that AlphaFold2 models with side-chain conformations differing from experimental structures yielded comparable hit rates, suggesting that productive docking conformations need not precisely match the crystallographic ground truth. This reinforces the rationale for selecting the docking model through empirical benchmarking with known ligands and decoys, rather than relying solely on structural accuracy metrics.

While our study utilized AlphaFold2 to generate the initial apo-kinase template, the fundamental principle of our ensemble-based docking workflow remains highly relevant and readily adaptable to newer structural prediction models, including AlphaFold3 [9]. The primary advantage of AlphaFold3 over AlphaFold2 lies in its diffusion-based architecture for predicting biomolecular complexes, including protein–ligand, protein–nucleic acid, and protein–protein interactions [9]. However, for protein monomer prediction, benchmarking studies have shown that AlphaFold3 offers only marginal improvements in global accuracy over AlphaFold2, with gains limited mainly to local structural quality [49]. Because our workflow uses the apo-form of the kinase domain as the starting template for ensemble generation, the AlphaFold3-predicted apo-structure is expected to differ minimally from that of AlphaFold2, and our conclusions regarding ensemble-based docking are therefore likely to remain applicable regardless of the AlphaFold version used. Nevertheless, the ability of AlphaFold3 to co-fold protein–ligand complexes may offer complementary opportunities for pose prediction and rescoring in future studies.

We are reluctant to assert that the existence of a new scaffold is a sufficient condition for selective inhibition. Potential off-targets cannot be predicted solely by comparing the chemical scaffolds of newly discovered and known inhibitors. The growth of bioactive molecules may increase the likelihood of discovering off-targets by comparing chemical similarities. However, considering the number of drug-like molecules that can exist, the proportion of currently available molecules is negligible in the chemical universe [50]. In such situations, the scarcity of similar bioactive molecules cannot guarantee their specificity [51]. Further experiments are required to fully characterize off-target liabilities. Nevertheless, novel chemotype inhibitors with altered specificity have been discovered, even in cases where several tens of thousands of LRRK2 inhibitors are known. The novel chemotype inhibitor identified in this study may provide new chemical tools and offer new opportunities for research on LRRK2-related diseases. Extensive hit-to-lead optimization was not performed within the scope of this study. The improvement in potency, selectivity, and pharmaceutical properties, including blood–brain barrier penetration via medicinal chemistry, along with orthogonal biochemical and biophysical validation, will be the focus of future studies.

## 4. Materials and Methods

### 4.1. Structural Ensemble Generation and Docking Protocol

A total of 3000 structural models of the LRRK2 kinase domain were generated using MODELLER (version 10.2) [52], based on the AlphaFold2-predicted structure as an initial template. MODELLER generates structures by combining conjugate gradients and molecular dynamics with simulated annealing, followed by the Discrete Optimized Protein Energy (DOPE). Of these, 500 models with the lowest DOPE scores were selected to construct a structural ensemble. All selected models were aligned to the AlphaFold2 structure using TM-align [53] to ensure a consistent orientation. Docking grids were generated for each structure using the standard protocol of Glide [54,55]. The protocol for preparing the ZINC library [56,57] was adapted to prepare mol2 files for docking with Glide-SP [54]. The protonation state of each molecule was calculated using cxcalc (ChemAxon, Budapest, Hungary), assuming a pH of 7.4. Subsequently, the data were converted into the mol2 format using molconvert.

The docking grid for Glide was centered on the centroid of the five putative contact residues (Lys1906, Glu1948, Ala1950, Asn1999, and Asp2017) identified in Section 2.1. The inner grid box was set to 10 Å × 10 Å × 10 Å, and the outer box was set to 30 Å × 30 Å × 30 Å, following the standard Glide protocol. Only Glide-SP (Standard Precision) was used; XP (Extra Precision) docking was not employed due to the computational cost of screening > 1 M compounds.

### 4.2. Docking-Based Model Selection and Dataset Preparation

The strongest 60 LRRK2 inhibitors in terms of pChEMBL value with molecular weights below 500 Da were retrieved from the ChEMBL database [25]. For each inhibitor, 50 physicochemically matched but topologically dissimilar decoys were generated using the DUD-E server [29]. These ligand–decoy sets were used to evaluate the enrichment capabilities of each model in the structural ensemble. Receiver operating characteristic (ROC) curves were constructed for each docking result, where the true and false positive rates were plotted on the y- and x-axes, respectively. The area under the curve (AUC) was computed for standard enrichment assessment [24]. For LogAUC calculation, the *x*-axis was logarithmically rescaled to the range of 10^−3^ to 1. The area under this transformed ROC curve was normalized by dividing by 3, and a baseline value of 0.145, representing random enrichment, was subtracted. The final AUC and LogAUC values were multiplied by 100 and expressed as percentages. Molecule preparation and docking screen followed the protocol described in Section 4.1. The resulting structures were used as inputs for docking simulations performed using the Glide-SP [54] algorithm.

### 4.3. High-Throughput Docking Screen

Approximately 1.3 million small molecules were obtained from the CORE and EXPRESS compound libraries provided by ChemBridge (San Diego, CA, USA). Docking simulations were automated using in-house Python scripts [5,6,58] and executed on a computing cluster with 100 CPU cores. All docking calculations were performed using the Glide-SP module (Schrödinger Suite 2022-3, New York, NY, USA). The intermolecular interactions between LRRK2 kinase and the docked ligands were analyzed using PyMOL (Schrödinger) and the OpenEye toolkit (OpenEye Scientific Software, Santa Fe, NM, USA).

Four criteria were used to filter out molecules with unsatisfactory contact. First, specific interactions with key residues were the primary features for candidate selection. Specific interactions with key residues, including hydrogen bonds and salt bridges, between the ligand and side chain were computationally identified using distance cut-offs (<3.5 Å). Molecules lacking all user-defined intermolecular hydrophilic interactions were excluded. Second, internal strain is often not included in energy functions but is an important concern. A computational tool that estimates torsion strain energies based on experimentally determined torsional populations was used to exclude molecules with high-energy torsion angles, following the recommendations of the creator of the tool [59]. Third, molecules with unsatisfactory hydrogen bond donors or acceptors, especially in the hydrophobic pockets of the site, were excluded when the number of unsatisfactory acceptors was >2 and the number of unsatisfactory donors was >0. Fourth, the docked poses of the remaining molecules were checked manually for the geometry of intermolecular hydrogen bonds and the overall complementarity between the ligand and the binding site.

### 4.4. Enzyme-Based Kinase Inhibition Assay

The compounds used in this study were purchased from Cayman Chemical (Ann Arbor, MI, USA), ChemBridge (San Diego, CA, USA), Enamine (Kyiv, Ukraine), and Sigma-Aldrich (St. Louis, MO, USA). Kinases and their corresponding substrates were obtained from SignalChem (Richmond, BC, Canada). Kinase activity was measured using the ADP-Glo^TM^ Kinase Assay (Promega, Madison, WI, USA) according to the manufacturer’s instructions. To evaluate assay-interference, each key compound was additionally tested in a detection-control format containing a fixed ATP/ADP mixture and the ADP-Glo detection reagents in the absence of LRRK2, confirming that none of the compounds altered the luminescence signal under the assay conditions. Reactions were performed in a buffer containing 40 mM Tris (pH 7.4), 20 mM MgCl_2_, and 0.01% Triton X-100. The final concentrations of the kinase, substrate, and ATP were 10 nM, 0.2 mg/mL, and 50 μM, respectively. For the LRRK2 assays, LRRKtide was used as the peptide substrate. The IC_50_ values were determined from the inhibitor concentration-dependent profile and converted to Ki values using the Cheng–Prusoff equation [60]. The Michaelis constant (Km) of ATP was determined from ATP titration experiments fitted to the Michaelis–Menten equation [61].

### 4.5. Cheminformatics

Cheminformatics analyses were performed using the RDKit. Extended connectivity fingerprints of radius 2 (ECFP4) with a 1024-bit length were used to calculate the pairwise chemical similarity. The Tanimoto coefficient (TC) was computed as the ratio of the intersection over the union of fingerprint bits between two molecules. A set of known human kinase inhibitors was retrieved from the ChEMBL v35 database [25] and used to exclude structurally similar molecules from the hit selection and quantify structural novelty.

## Figures and Tables

**Table 1:** Chemical structures, docking rankings, and inhibitory activities of hit compounds.

ID	Chemical Vendor ID	Rank	Glide-SP (kcal/mol)	MW cLogP	K_i_ (μM)	Cloest Known LRRK2 Inhibitors ChEMBL ID (Tanimoto Coefficient)
**4**	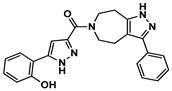 56275466	29	−9.46	399 1.6	6.3	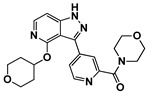 CHEMBL3651748 (0.282)
**14**	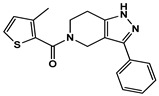 29661093	195	−9.05	323 3.6	10.3	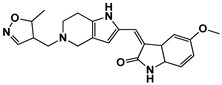 CHEMBL2403368 (0.273)
**15**	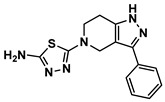 21393542	199	−9.05	298 1.3	0.5	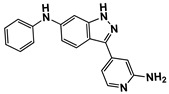 CHEMBL4060479 (0.262)
**16**	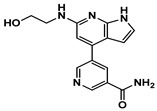 99611978	333	−8.94	297 0.3	1.4	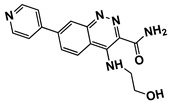 CHEMBL233139 (0.333)

ID in this study, vendor ID (ChemBridge), docked ranking and score, molecular weight, calculated LogP, Ki value, and closest known LRRK2 inhibitor with Tanimoto coefficient are shown for each compound.

**Table 2:** Chemical structures and inhibitory activities of analogs-by-catalog derived from compound **15**.

ID	Vendor ID	MW	cLogP	K_i_ (µM)
**21**	67665493	385	1.9	21.8
**22**	35007010	374	3.0	17.4
**23**	67546378	380	3.5	37.7
**24**	94709025	395	3.6	3.8
**25**	93174098	392	0.6	141.6
**26**	62023574	383	2.2	189.4
**27**	74985664	385	2.2	3.1
**28**	21674932	374	1.2	87.7
**29**	76291233	380	-0.9	14.2
**30**	90612118	395	2.8	22.4
**31**	84539367	392	1.8	413.3
**32**	89410380	383	1.9	18.7
**33**	Z3831423838	379	1.9	1.3
**34**	Z3831423115	374	1.9	3.1
**35**	PV-003231882198	361	0.4	22.0
**36**	Z3831422674	351	1.9	7.1
**38**	PV-003514523304	347	0.9	9.47
**39**	Z3831425106	339	1.6	61.72
**40**	Z3831422601	347	1.3	19.68
**41**	Z3831422458	331	1.6	7.14
**42**	Z3831422390	345	2.1	25.52

ID in this study, vendor ID from either ChemBridge (**21**–**32**) or Enamine (**33**–**42**), molecular weight, calculated LogP, and Ki values are shown.

**Table 3:** Selectivity profiling of compounds **15**, **16**, and **33** against a panel of kinases.

ID	LRRK2 (G2019S)	MST1	CLK2	TTK	ASK1	NIK	SYK
**15**	0.8	13.9	0.7	1.8	86.6	26.2	155.6
**16**	2.7	32.6	22.3	4.0	0.9	45.1	277.7
**33**	1.2	0.9	26.1	30.1	152.3	130.6	91.5

Determined Ki values (μM) for LRRK2 (G2019S), MST1, CLK2, TTK, ASK1, NIK, and SYK are shown. Values below 5 μM are highlighted in red.

## Data Availability

The original contributions presented in this study are included in the article/Supplementary Materials. Further inquiries can be directed to the corresponding author.

## Supplementary Materials

The following supporting information can be downloaded at: https://www.mdpi.com/article/10.3390/ijms27083391/s1.

## References

[1] Shoichet B.K. (2004). Virtual screening of chemical libraries. Nature.

[2] Dang C.V., Reddy E.P., Shokat K.M., Soucek L. (2017). Drugging the ‘undruggable’ cancer targets. Nat. Rev. Cancer.

[3] Irwin J.J., Shoichet B.K. (2016). Docking screens for novel ligands conferring new biology: Miniperspective. J. Med. Chem..

[4] Miller E.B., Murphy R.B., Sindhikara D., Borrelli K.W., Grisewood M.J., Ranalli F., Dixon S.L., Jerome S., Boyles N.A., Day T. (2021). Reliable and Accurate Solution to the Induced Fit Docking Problem for Protein-Ligand Binding. J. Chem. Theory Comput..

[5] Choi J., Choi K.E., Park S.J., Kim S.Y., Jee J.G. (2016). Ensemble-Based Virtual Screening Led to the Discovery of New Classes of Potent Tyrosinase Inhibitors. J. Chem. Inf. Model..

[6] Kim H.H., Hyun J.S., Choi J., Choi K.E., Jee J.G., Park S.J. (2018). Structural ensemble-based docking simulation and biophysical studies discovered new inhibitors of Hsp90 N-terminal domain. Sci. Rep..

[7] Jumper J., Evans R., Pritzel A., Green T., Figurnov M., Ronneberger O., Tunyasuvunakool K., Bates R., Zidek A., Potapenko A. (2021). Highly accurate protein structure prediction with AlphaFold. Nature.

[8] Tunyasuvunakool K., Adler J., Wu Z., Green T., Zielinski M., Zidek A., Bridgland A., Cowie A., Meyer C., Laydon A. (2021). Highly accurate protein structure prediction for the human proteome. Nature.

[9] Abramson J., Adler J., Dunger J., Evans R., Green T., Pritzel A., Ronneberger O., Willmore L., Ballard A.J., Bambrick J. (2024). Accurate structure prediction of biomolecular interactions with AlphaFold 3. Nature.

[10] Tolosa E., Vila M., Klein C., Rascol O. (2020). LRRK2 in Parkinson disease: Challenges of clinical trials. Nat. Rev. Neurol..

[11] Deniston C.K., Salogiannis J., Mathea S., Snead D.M., Lahiri I., Matyszewski M., Donosa O., Watanabe R., Bohning J., Shiau A.K. (2020). Structure of LRRK2 in Parkinson’s disease and model for microtubule interaction. Nature.

[12] Watanabe R., Buschauer R., Bohning J., Audagnotto M., Lasker K., Lu T.W., Boassa D., Taylor S., Villa E. (2020). The In Situ Structure of Parkinson’s Disease-Linked LRRK2. Cell.

[13] Gilsbach B.K., Ho F.Y., Vetter I.R., van Haastert P.J., Wittinghofer A., Kortholt A. (2012). Roco kinase structures give insights into the mechanism of Parkinson disease-related leucine-rich-repeat kinase 2 mutations. Proc. Natl. Acad. Sci. USA.

[14] Gilsbach B.K., Messias A.C., Ito G., Sattler M., Alessi D.R., Wittinghofer A., Kortholt A. (2015). Structural Characterization of LRRK2 Inhibitors. J. Med. Chem..

[15] Williamson D.S., Smith G.P., Mikkelsen G.K., Jensen T., Acheson-Dossang P., Badolo L., Bedford S.T., Chell V., Chen I.J., Dokurno P. (2021). Design and Synthesis of Pyrrolo[2,3-d]pyrimidine-Derived Leucine-Rich Repeat Kinase 2 (LRRK2) Inhibitors Using a Checkpoint Kinase 1 (CHK1)-Derived Crystallographic Surrogate. J. Med. Chem..

[16] Williamson D.S., Smith G.P., Acheson-Dossang P., Bedford S.T., Chell V., Chen I.J., Daechsel J.C.A., Daniels Z., David L., Dokurno P. (2017). Design of Leucine-Rich Repeat Kinase 2 (LRRK2) Inhibitors Using a Crystallographic Surrogate Derived from Checkpoint Kinase 1 (CHK1). J. Med. Chem..

[17] Zhu H., Hixson P., Ma W., Sun J. (2024). Pharmacology of LRRK2 with type I and II kinase inhibitors revealed by cryo-EM. Cell Discov..

[18] Healy D.G., Falchi M., O’Sullivan S.S., Bonifati V., Durr A., Bressman S., Brice A., Aasly J., Zabetian C.P., Goldwurm S. (2008). Phenotype, genotype, and worldwide genetic penetrance of LRRK2-associated Parkinson’s disease: A case-control study. Lancet Neurol..

[19] Fell M.J., Mirescu C., Basu K., Cheewatrakoolpong B., DeMong D.E., Ellis J.M., Hyde L.A., Lin Y., Markgraf C.G., Mei H. (2015). MLi-2, a Potent, Selective, and Centrally Active Compound for Exploring the Therapeutic Potential and Safety of LRRK2 Kinase Inhibition. J. Pharmacol. Exp. Ther..

[20] Henderson J.L., Kormos B.L., Hayward M.M., Coffman K.J., Jasti J., Kurumbail R.G., Wager T.T., Verhoest P.R., Noell G.S., Chen Y. (2015). Discovery and preclinical profiling of 3-[4-(morpholin-4-yl)-7*H*-pyrrolo[2,3-*d*] pyrimidin-5-yl]benzonitrile (PF-06447475), a highly potent, selective, brain penetrant, and in vivo active LRRK2 kinase inhibitor. J. Med. Chem..

[21] Estrada A.A., Chan B.K., Baker-Glenn C., Beresford A., Burdick D.J., Chambers M., Chen H., Dominguez S.L., Dotson J., Drummond J. (2014). Discovery of highly potent, selective, and brain-penetrant aminopyrazole leucine-rich repeat kinase 2 (LRRK2) small molecule inhibitors. J. Med. Chem..

[22] Jennings D., Huntwork-Rodriguez S., Vissers M., Daryani V.M., Diaz D., Goo M.S., Chen J.J., Maciuca R., Fraser K., Mabrouk O.S. (2023). LRRK2 Inhibition by BIIB122 in Healthy Participants and Patients with Parkinson’s Disease. Mov. Disord..

[23] Holm L., Rosenstrom P. (2010). Dali server: Conservation mapping in 3D. Nucleic Acids Res..

[24] Mysinger M.M., Shoichet B.K. (2010). Rapid context-dependent ligand desolvation in molecular docking. J. Chem. Inf. Model..

[25] Mendez D., Gaulton A., Bento A.P., Chambers J., De Veij M., Felix E., Magarinos M.P., Mosquera J.F., Mutowo P., Nowotka M. (2019). ChEMBL: Towards direct deposition of bioassay data. Nucleic Acids Res..

[26] Lo Y.C., Senese S., Damoiseaux R., Torres J.Z. (2016). 3D Chemical Similarity Networks for Structure-Based Target Prediction and Scaffold Hopping. ACS Chem. Biol..

[27] Yang R., Zhou H., Wang F., Yang G. (2024). DigFrag as a digital fragmentation method used for artificial intelligence-based drug design. Commun. Chem..

[28] Zhou G., Rusnac D.V., Park H., Canzani D., Nguyen H.M., Stewart L., Bush M.F., Nguyen P.T., Wulff H., Yarov-Yarovoy V. (2024). An artificial intelligence accelerated virtual screening platform for drug discovery. Nat. Commun..

[29] Mysinger M.M., Carchia M., Irwin J.J., Shoichet B.K. (2012). Directory of useful decoys, enhanced (DUD-E): Better ligands and decoys for better benchmarking. J. Med. Chem..

[30] Bender B.J., Gahbauer S., Luttens A., Lyu J., Webb C.M., Stein R.M., Fink E.A., Balius T.E., Carlsson J., Irwin J.J. (2021). A practical guide to large-scale docking. Nat. Protoc..

[31] Feng B.Y., Shoichet B.K. (2006). A detergent-based assay for the detection of promiscuous inhibitors. Nat. Protoc..

[32] Daina A., Michielin O., Zoete V. (2017). SwissADME: A free web tool to evaluate pharmacokinetics, drug-likeness and medicinal chemistry friendliness of small molecules. Sci. Rep..

[33] Passaro S., Corso G., Wohlwend J., Reveiz M., Thaler S., Somnath V.R., Getz N., Portnoi T., Roy J., Stark H. (2025). Boltz-2: Towards Accurate and Efficient Binding Affinity Prediction. bioRxiv.

[34] Lounkine E., Keiser M.J., Whitebread S., Mikhailov D., Hamon J., Jenkins J.L., Lavan P., Weber E., Doak A.K., Cote S. (2012). Large-scale prediction and testing of drug activity on side-effect targets. Nature.

[35] Keiser M.J., Roth B.L., Armbruster B.N., Ernsberger P., Irwin J.J., Shoichet B.K. (2007). Relating protein pharmacology by ligand chemistry. Nat. Biotechnol..

[36] Conway P., Tyka M.D., DiMaio F., Konerding D.E., Baker D. (2014). Relaxation of backbone bond geometry improves protein energy landscape modeling. Protein Sci..

[37] Yang Q., Sharp K.A. (2009). Building alternate protein structures using the elastic network model. Proteins.

[38] Mirdita M., Schutze K., Moriwaki Y., Heo L., Ovchinnikov S., Steinegger M. (2022). ColabFold: Making protein folding accessible to all. Nat. Methods.

[39] Diaz-Rovira A.M., Martin H., Beuming T., Diaz L., Guallar V., Ray S.S. (2023). Are Deep Learning Structural Models Sufficiently Accurate for Virtual Screening? Application of Docking Algorithms to AlphaFold2 Predicted Structures. J. Chem. Inf. Model..

[40] Scardino V., Di Filippo J.I., Cavasotto C.N. (2023). How good are AlphaFold models for docking-based virtual screening?. iScience.

[41] Lyu J., Kapolka N., Gumpper R., Alon A., Wang L., Jain M.K., Barros-Alvarez X., Sakamoto K., Kim Y., DiBerto J. (2024). AlphaFold2 structures guide prospective ligand discovery. Science.

[42] Ren F., Ding X., Zheng M., Korzinkin M., Cai X., Zhu W., Mantsyzov A., Aliper A., Aladinskiy V., Cao Z. (2023). AlphaFold accelerates artificial intelligence powered drug discovery: Efficient discovery of a novel CDK20 small molecule inhibitor. Chem. Sci..

[43] Baselious F., Hilscher S., Robaa D., Barinka C., Schutkowski M., Sippl W. (2024). Comparative Structure-Based Virtual Screening Utilizing Optimized AlphaFold Model Identifies Selective HDAC11 Inhibitor. Int. J. Mol. Sci..

[44] Diaz-Holguin A., Saarinen M., Vo D.D., Sturchio A., Branzell N., Cabeza de Vaca I., Hu H., Mitjavila-Domenech N., Lindqvist A., Baranczewski P. (2024). AlphaFold accelerated discovery of psychotropic agonists targeting the trace amine-associated receptor 1. Sci. Adv..

[45] Modi V., Dunbrack R.L. (2019). Defining a new nomenclature for the structures of active and inactive kinases. Proc. Natl. Acad. Sci. USA.

[46] Song J., Ha J., Lee J., Ko J., Shin W.H. (2024). Improving docking and virtual screening performance using AlphaFold2 multi-state modeling for kinases. Sci. Rep..

[47] Irwin J.J., Shoichet B.K., Mysinger M.M., Huang N., Colizzi F., Wassam P., Cao Y. (2009). Automated docking screens: A feasibility study. J. Med. Chem..

[48] Sanz Murillo M., Villagran Suarez A., Dederer V., Chatterjee D., Alegrio Louro J., Knapp S., Mathea S., Leschziner A.E. (2023). Inhibition of Parkinson’s disease-related LRRK2 by type I and type II kinase inhibitors: Activity and structures. Sci. Adv..

[49] Peng C., Ni W., Liu Q., Hu G., Zheng W. (2025). A comprehensive benchmarking of the AlphaFold3 for predicting biomacromolecules and their interactions. Brief. Bioinform..

[50] Mullard A. (2017). The Drug-Maker’s Guide to the Galaxy. Nature.

[51] Hert J., Irwin J.J., Laggner C., Keiser M.J., Shoichet B.K. (2009). Quantifying biogenic bias in screening libraries. Nat. Chem. Biol..

[52] Marti-Renom M.A., Stuart A.C., Fiser A., Sanchez R., Melo F., Sali A. (2000). Comparative protein structure modeling of genes and genomes. Annu. Rev. Biophys. Biomol. Struct..

[53] Zhang Y., Skolnick J. (2005). TM-align: A protein structure alignment algorithm based on the TM-score. Nucleic Acids Res..

[54] Halgren T.A., Murphy R.B., Friesner R.A., Beard H.S., Frye L.L., Pollard W.T., Banks J.L. (2004). Glide: A new approach for rapid, accurate docking and scoring. 2. Enrichment factors in database screening. J. Med. Chem..

[55] Friesner R.A., Murphy R.B., Repasky M.P., Frye L.L., Greenwood J.R., Halgren T.A., Sanschagrin P.C., Mainz D.T. (2006). Extra precision glide: Docking and scoring incorporating a model of hydrophobic enclosure for protein-ligand complexes. J. Med. Chem..

[56] Irwin J.J., Sterling T., Mysinger M.M., Bolstad E.S., Coleman R.G. (2012). ZINC: A free tool to discover chemistry for biology. J. Chem. Inf. Model..

[57] Irwin J.J., Tang K.G., Young J., Dandarchuluun C., Wong B.R., Khurelbaatar M., Moroz Y.S., Mayfield J., Sayle R.A. (2020). ZINC20-A Free Ultralarge-Scale Chemical Database for Ligand Discovery. J. Chem. Inf. Model..

[58] Lee W., Lee S., Choi J., Park J.H., Kim K.M., Jee J.G., Bae J.S. (2017). Antithrombotic properties of JJ1, a potent and novel thrombin inhibitor. Sci. Rep..

[59] Gu S., Smith M.S., Yang Y., Irwin J.J., Shoichet B.K. (2021). Ligand Strain Energy in Large Library Docking. J. Chem. Inf. Model..

[60] Cornish-Bowden A. (2013). Fundamentals of Enzyme Kinetics.

[61] Srinivasan B. (2022). A guide to the Michaelis-Menten equation: Steady state and beyond. FEBS J..

