# Supplementary material for: Discovery of Novel Chemotype LRRK2 Inhibitors Through AlphaFold2-Generated Structure-Based Docking Screen
Source: Int J Mol Sci. 2026 Apr 9;27(8):3391. doi: 10.3390/ijms27083391 (PMC13116677; doi:10.3390/ijms27083391)
Supplement: Supplementary file 1 [file ijms-27-03391-s001.zip › Supplementary Materials Figures S1~S9.pdf]

Supplementary materials

Discovery of Novel Chemotype LRRK2 Inhibitors through AlphaFold2-Generated Structure-Based Docking Screen

Rishiram Baral†, Jeong In Lee†, and Jun-Goo Jee*

Research Institute of Pharmaceutical Sciences, College of Pharmacy, Kyungpook National University, Daegu, Republic of Korea

* Corresponding author: jjee@knu.ac.kr

†These authors contributed equally to this work.

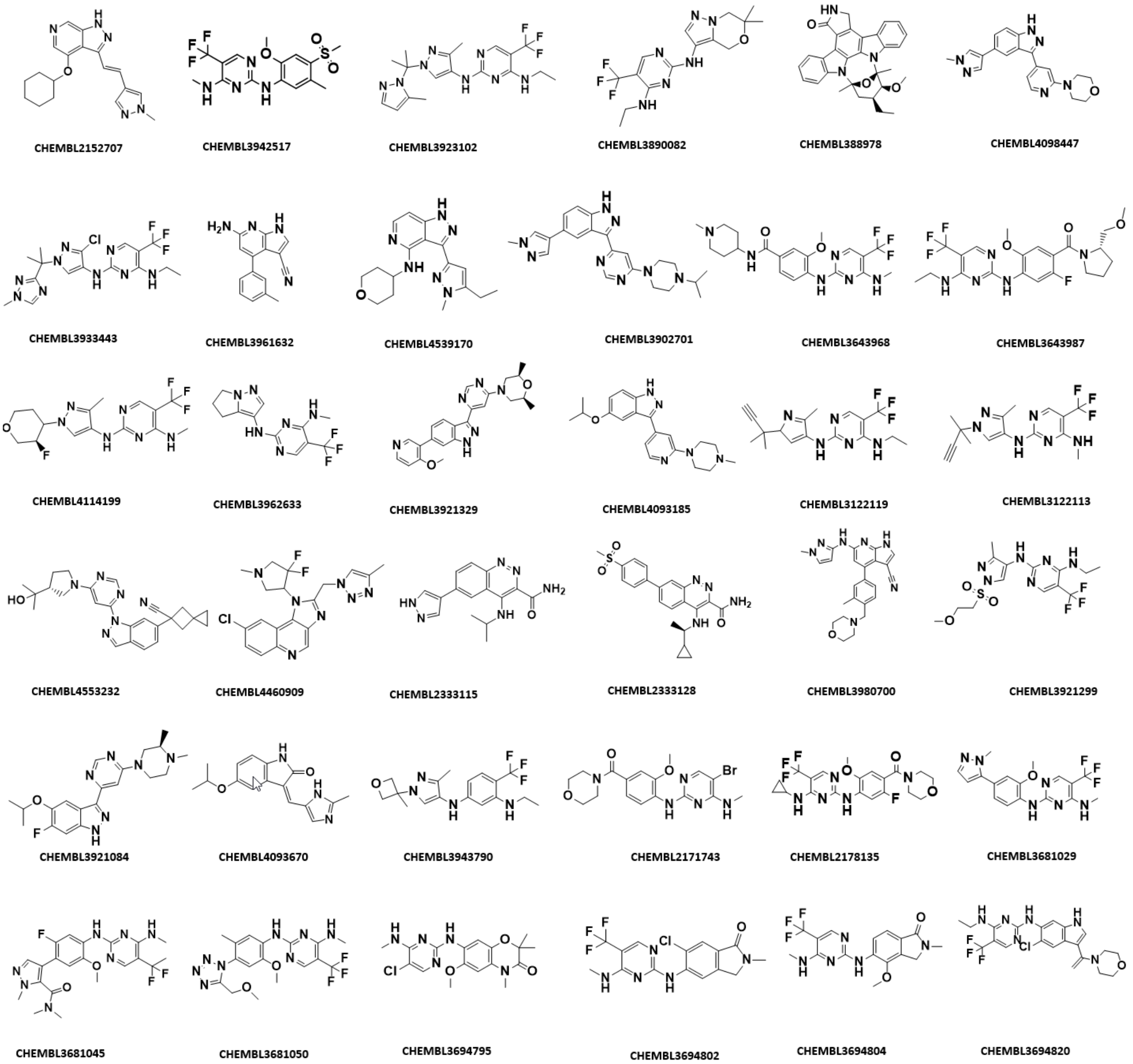

Figure S1. Selected reference inhibitors used for docking-based model evaluation. Thirty-six known LRRK2 inhibitors selected from the ChEMBL database were used to evaluate the enrichment performance of each conformer in the docking-based model-selection step. Molecules were used as true-positive ligands for calculating the AUC and LogAUC metrics during the ROC-based evaluation.

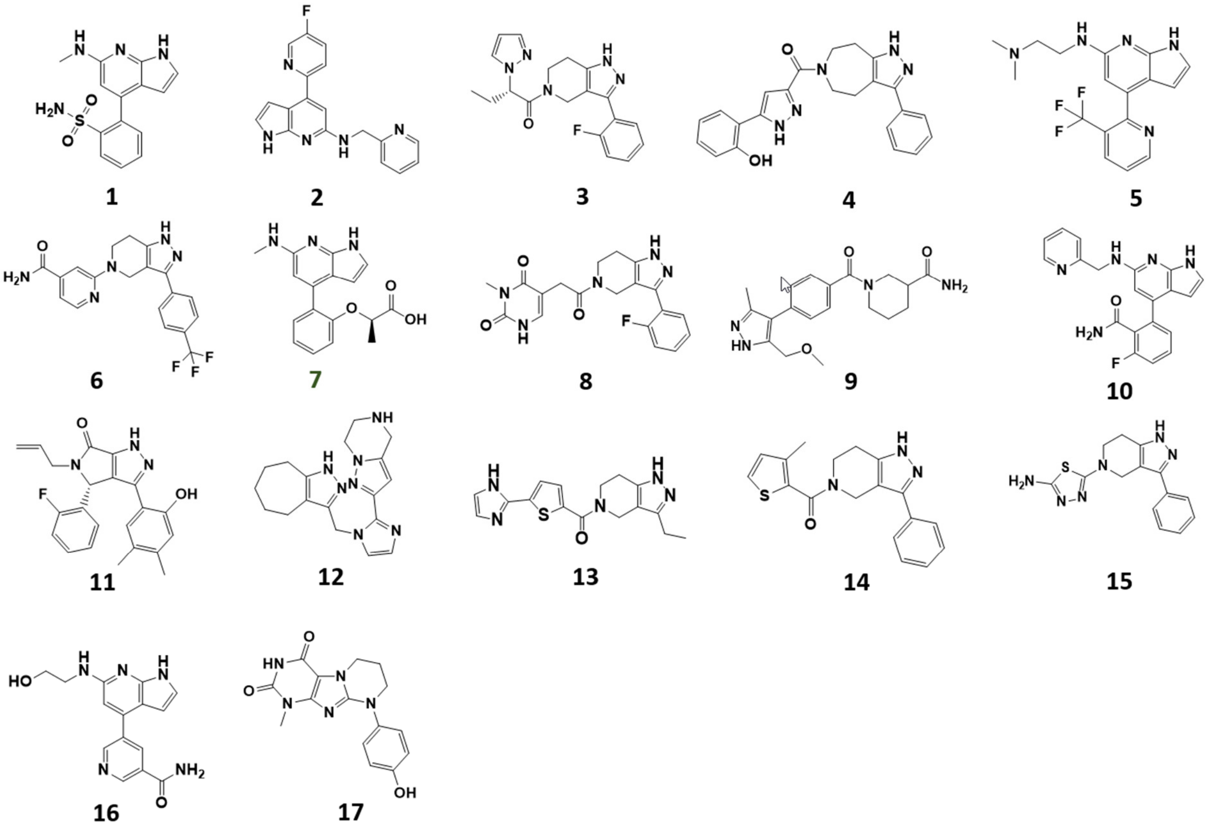

Figure S2. Chemical structures of the 17 candidate compounds selected from the docking screen. Seventeen small molecules were selected from a library of approximately 1.3 million compounds based on their docking scores and post-docking filtering criteria, including physicochemical properties, key interactions, and structural diversity of the compounds. These candidates were subjected to biochemical assays to assess their kinase inhibition.

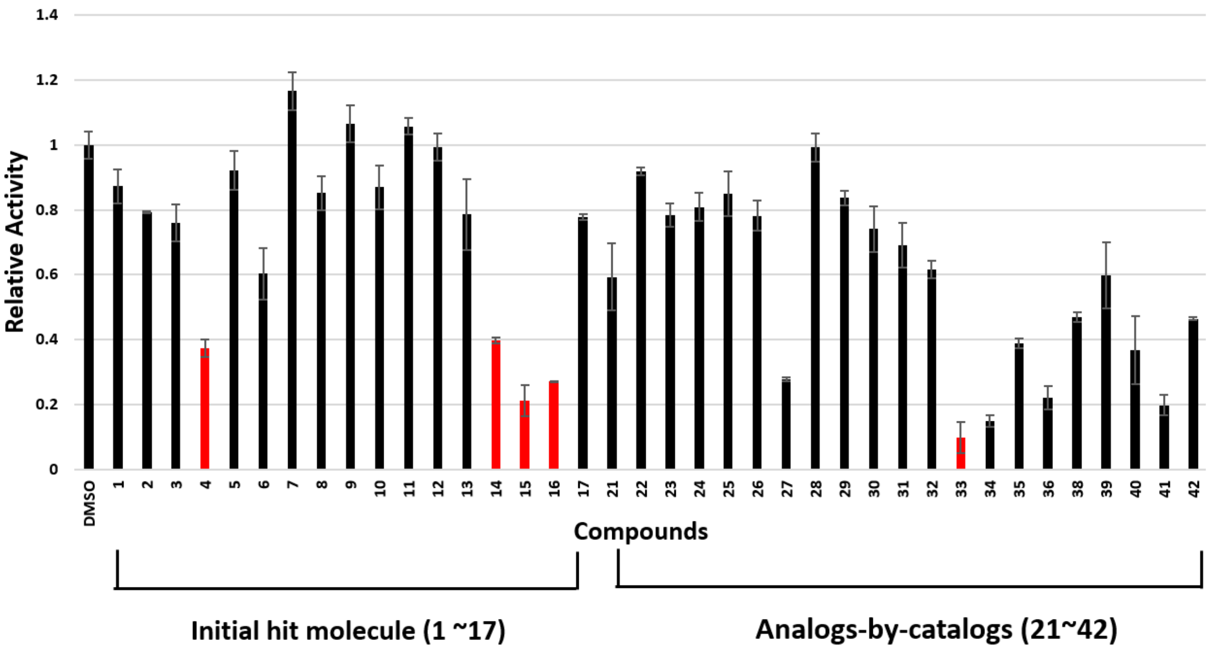

Figure S3. Inhibitory effects of initial hit compounds (**1–17**) and analogs of compound **15** (**21–42**). Relative kinase activities were measured in the presence of each compound at a concentration of 50 μM. Red bars highlight the compounds that showed a notable reduction in kinase activity compared to the DMSO control. Error bars represent the standard deviation of duplicate measurements.

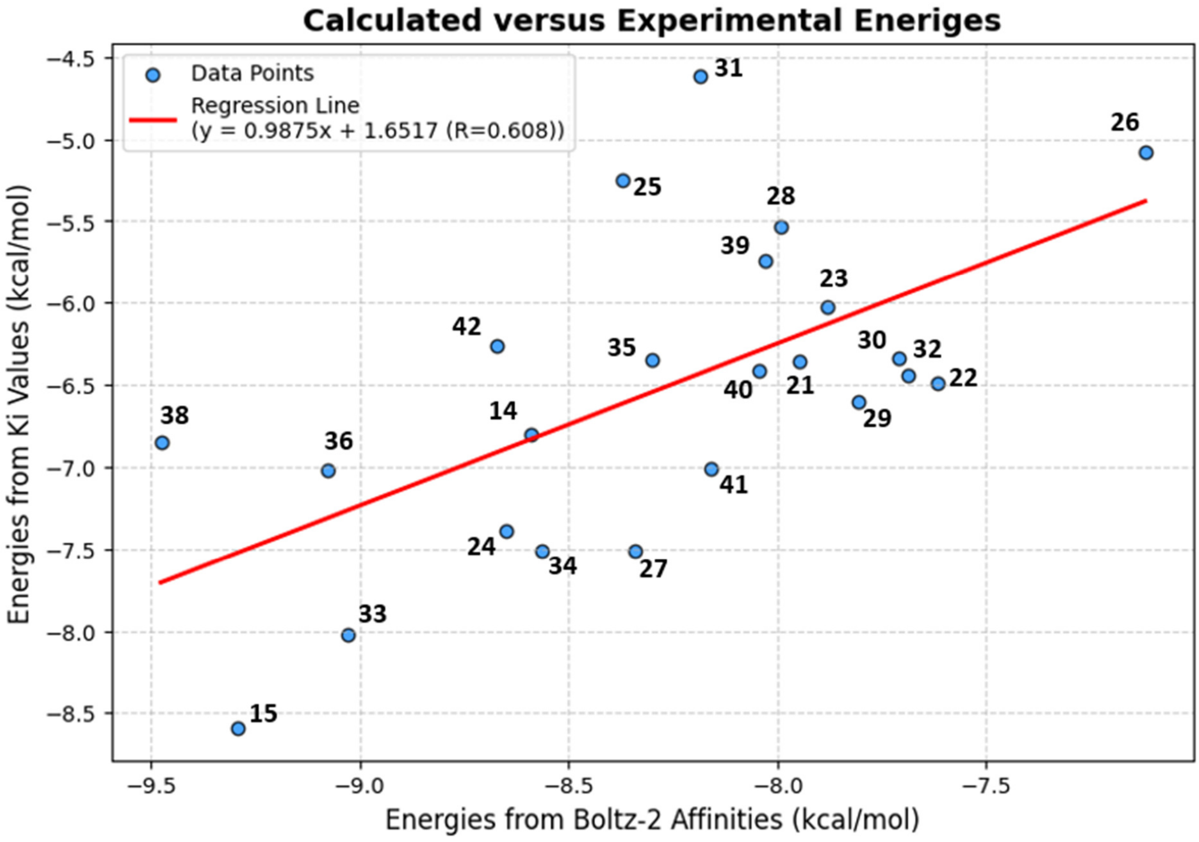

Figure S4. Comparison of experimentally estimated and computationally calculated free energies. The ID of each molecule is labeled for each data point. After the co-folding of each molecule (**14**, **15**, and **21**–**42**) with the LRRK2 kinase domain using Boltz-2, the affinity extraction in the best complex model of the five runs was performed. The Ki value (M) was converted into free energy (kcal/mol) using the formula "free energy = $1.363\times\mathrm{Log}_{10}$[Ki]". The probability that this correlation occurs by chance is 2.1×10^{-3}, indicating that Boltz-2 can quantitatively predict these affinities.

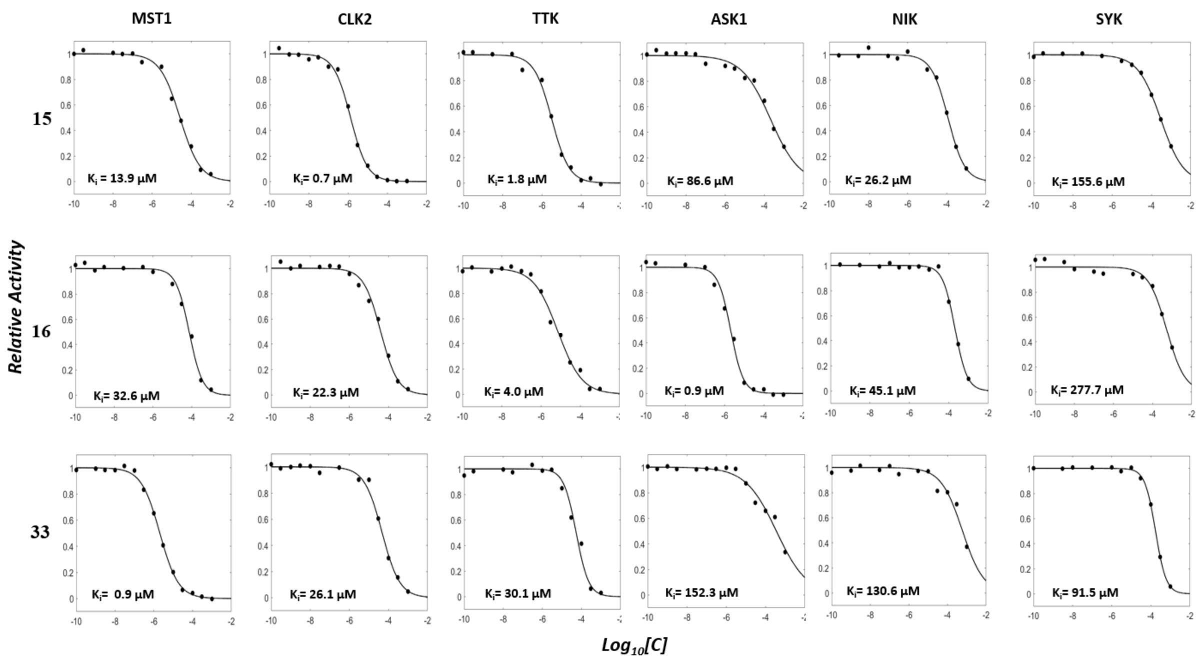

Figure S5. Concentration-dependent inhibition profiles of selected compounds on off-target kinases. Compounds **15**, **16**, and **33** were profiled against six kinases (MST1, CLK2, TTK, ASK1, NIK, and SYK) to assess their potential off-target activity. These kinases were selected based on prior reports of off-target inhibition by known LRRK2 inhibitors or their structural similarity to the LRRK2 kinase domain. Relative kinase activity was measured across a range of compound concentrations, and the corresponding Ki values are indicated.

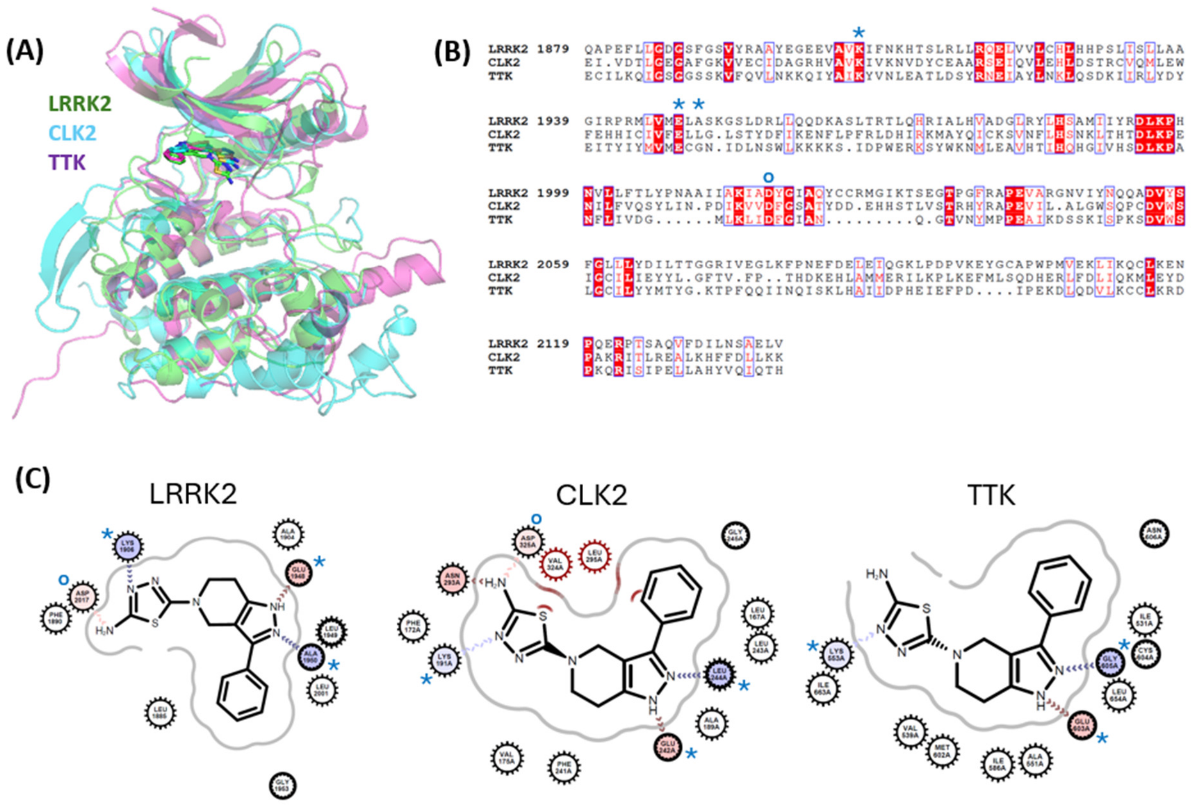

Figure S6. Comparison of the interactions between **15** and LRRK2, **15** and CLK2, and **15** and TTK. (A) Structural comparison of the complex structures between **15** and LRRK2, **15** and CLK2, and **15** and TTK kinase domains. (B) Structure-based sequence alignment of LRRK2, CLK2, and TTK kinase domains. (C) 2D diagrams for intermolecular contacts between **15** and LRRK2, CLK2, TTK kinase domains. The residues found in all three proteins are labeled with *, and those found in the two proteins are labeled with o in (B) and (C).

Query	Target Key	Target Name	Description	P-Value	MaxTC
**15** 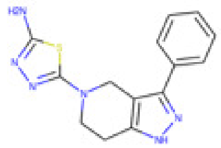 compound_1	MK01_MOUSE	Mapk1	Mitogen-activated protein kinase 1	3.575e-14	0.36
	5HT1E_HUMAN	HTR1E	5-hydroxytryptamine receptor 1E	2.042e-06	0.33

Query	Target Key	Target Name	Description	P-Value	MaxTC
**16** 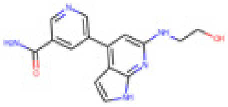 compound_1	NNMT_MOUSE	Nnmt	Nicotinamide N-methyltransferase	3.875e-36	0.29
	SIR3_HUMAN	SIRT3	NAD-dependent protein deacetylase sirtuin-3, mitochondrial	2.39e-28	0.36
	SIR2_HUMAN	SIRT2	NAD-dependent protein deacetylase sirtuin-2	1.41e-19	0.36
	HGFL_HUMAN	MST1	Hepatocyte growth factor-like protein	2.898e-18	0.29
	SIR1_HUMAN	SIRT1	NAD-dependent protein deacetylase sirtuin-1	4.596e-14	0.34
	NNMT_HUMAN	NNMT	Nicotinamide N-methyltransferase	9.7e-12	0.29
	HPGDS_HUMAN	HPGDS	Hematopoietic prostaglandin D synthase	6.713e-11	0.34
	8ODP_HUMAN	NUDT1	7,8-dihydro-8-oxoguanine triphosphatase	1.825e-08	0.33
	MK01_RAT	Mapk1	Mitogen-activated protein kinase 1	3.985e-08	0.29
	M4K4_HUMAN	MAP4K4	Mitogen-activated protein kinase kinase kinase kinase 4	4.386e-08	0.36
	GYRB_STAAU	gyrB	DNA gyrase subunit B	2.685e-06	0.36
	FES_HUMAN	FES	Tyrosine-protein kinase Fes/Fps	2.971e-06	0.32
	PASK_HUMAN	PASK	PAS domain-containing serine/threonine-protein kinase	4.478e-06	0.30
	ST17A_HUMAN	STK17A	Serine/threonine-protein kinase 17A	9.329e-06	0.33
	SGK2_HUMAN	SGK2	Serine/threonine-protein kinase Sgk2	9.979e-06	0.33

Query	Target Key	Target Name	Description	P-Value	MaxTC
**33** 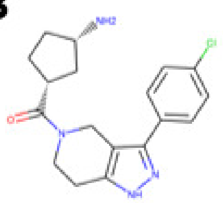 compound_1	RET4_HUMAN	RBP4	Retinol-binding protein 4	2.189e-48	0.34
	KCNK3_HUMAN	KCNK3	Potassium channel subfamily K member 3	7.725e-22	0.39
	MK11_HUMAN	MAPK11	Mitogen-activated protein kinase 11	1.334e-19	0.37
	MK01_MOUSE	Mapk1	Mitogen-activated protein kinase 1	1.262e-18	0.42
	TKNK_HUMAN	TAC3	Tachykinin-3	8.289e-12	0.29
	SIR1_HUMAN	SIRT1	NAD-dependent protein deacetylase sirtuin-1	6.861e-09	0.44
	AOFB_BOVIN	MAOB	Amine oxidase [flavin-containing] B	1.176e-08	0.29
	P2RX7_RAT	P2rx7	P2X purinoceptor 7	5.249e-08	0.41
	5HT1E_HUMAN	HTR1E	5-hydroxytryptamine receptor 1E	3.688e-07	0.43
	AOFA_BOVIN	MAOA	Amine oxidase [flavin-containing] A	3.68e-06	0.29
	CCNC_HUMAN	CCNC	Cyclin-C	3.774e-06	0.32

Figure S7. Similarity Ensemble Approach using **15**, **16**, and **33.** SEA server (sea.docking.org) was used.

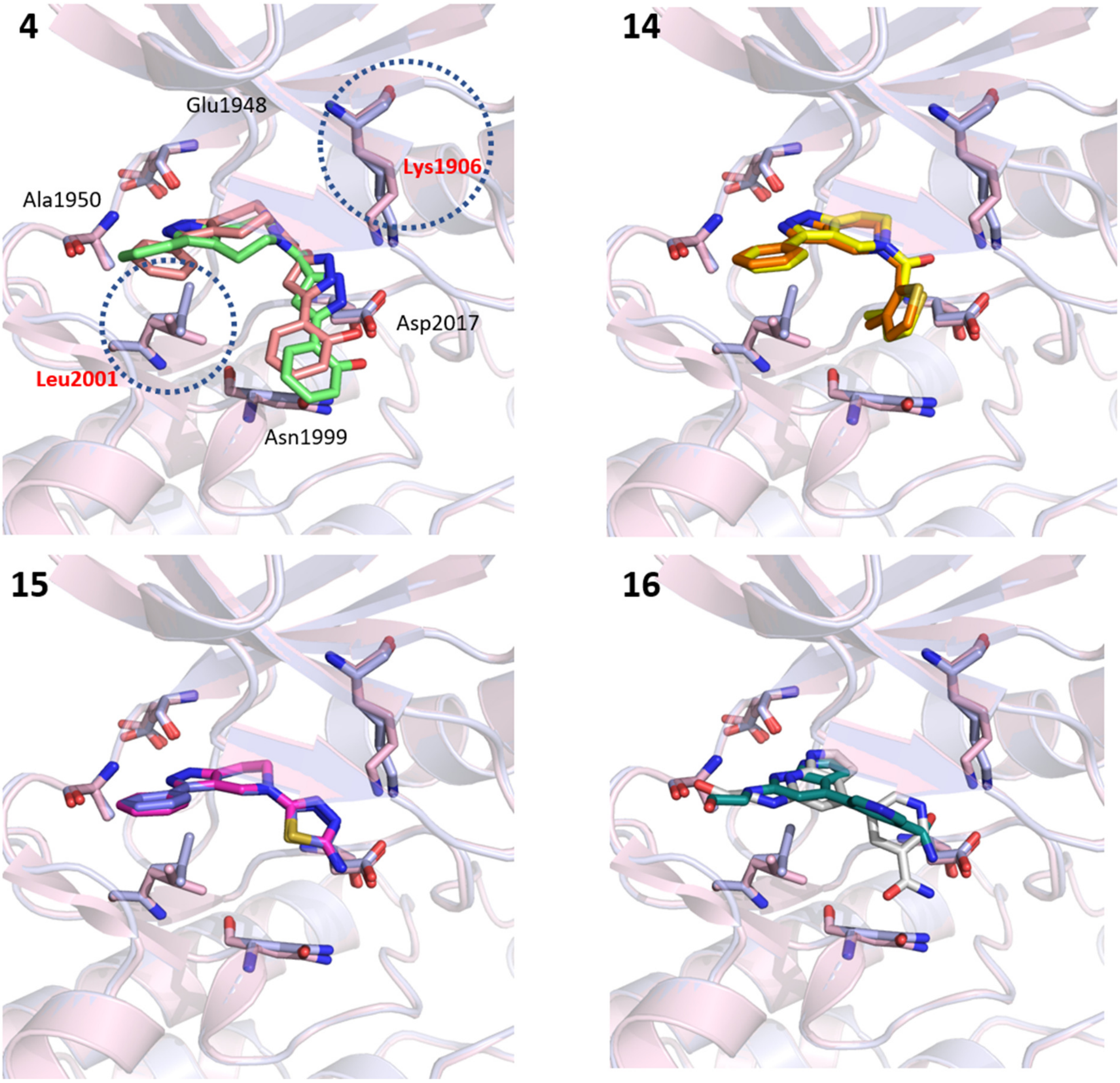

Figure S8. Structural comparison of ligand binding poses between AlphaFold2-predicted and ensemble-derived models. Docked poses of compounds **4**, **14**, **15**, and **16** are shown in superposition between AlphaFold2-generated and ensemble-derived structures, aligned consistently with the orientation in Figure 4 of the main text. Notably, Lys1906 and Leu2001 exhibit substantial positional deviations between the two structural models, as highlighted with dashed circles in the compound 4 panel.

(A)

	AlphaFold2	**0360**	8FO7	8U7H	8TXZ
AlphaFold2	0	0.9986	0.9692	0.9605	0.963
**0360**	0.2268	0	0.9673	0.9584	0.9605
8FO7	1.1128	1.1471	0	0.9874	0.9816
8U7H	1.2857	1.3207	0.689	0	0.9807
8TXZ	1.2556	1.2954	0.8335	0.8518	0

(B)

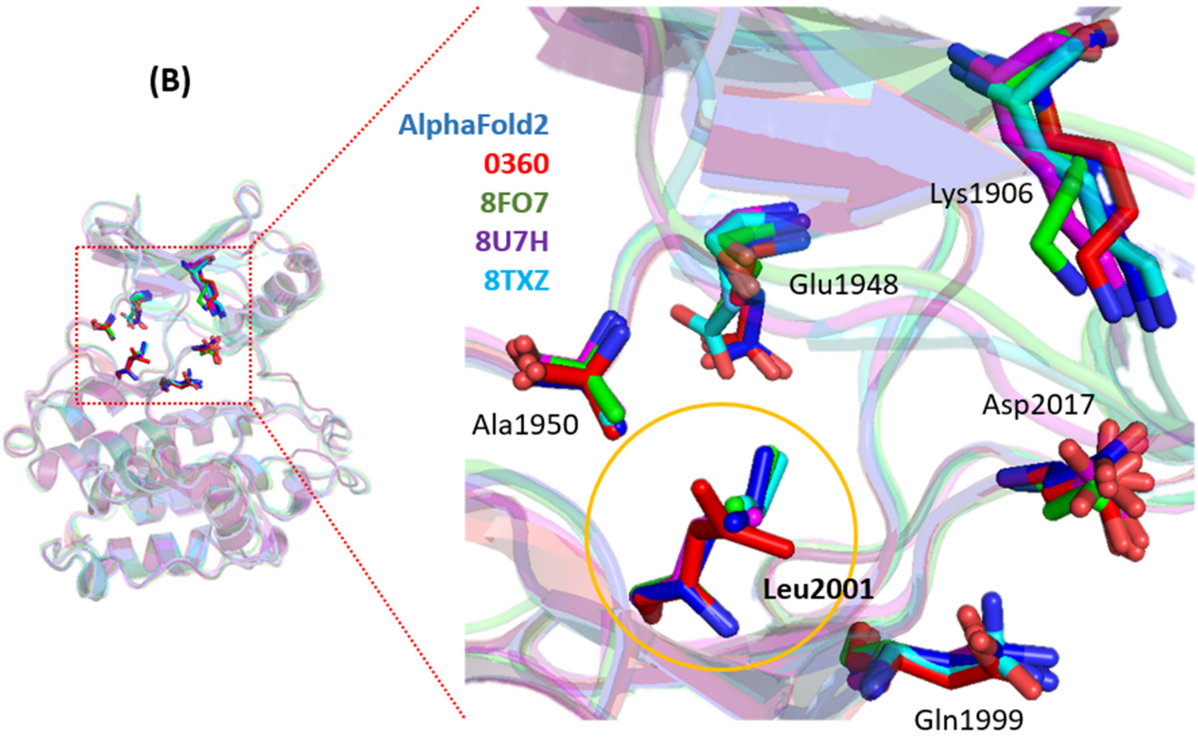

Figure S9. Structural superposition of conformer 0360 with AlphaFold2 template and cryo-EM structures of the LRRK2 kinase domain bound to type I inhibitors. (A) Quantified similarities between conformer 0360 for docking screen in this study, AlphaFold2 template, 8FO7 (PDB ID for the LRRK2 complex), 8U7H, and 8TXZ. The left side of the diagonal contains the pairwise backbone RMSDs with the unit of Å. The right side of the diagonal shows the pairwise TM-scores. (B) Overlaid structures of conformer 0360, AlphaFold2 template, 8FO7, 8U7H, and 8TXZ are shown (left). An enlarged view of the ATP-binding site with key residues is shown (right). The corresponding colors of the PDBs are blue for AlphaFold2 template, red for 0360, green for 8FO7, violet for 8U7H, and cyan for 8TXZ. The most altered residue, Leu2001, is circled in yellow.
